# Quality Assessment of CEUS in Individuals with Small Renal Masses—Which Individual Factors Are Associated with High Image Quality?

**DOI:** 10.3390/jcm9124081

**Published:** 2020-12-17

**Authors:** Paul Spiesecke, Thomas Fischer, Frank Friedersdorff, Bernd Hamm, Markus Herbert Lerchbaumer

**Affiliations:** 1 Department of Radiology, Charité-Universitätsmedizin Berlin, 10117 Berlin, Germany; paul.spiesecke@charite.de (P.S.); thom.fischer@charite.de (T.F.); bernd.hamm@charite.de (B.H.); 2 Department of Urology, Charité-Universitätsmedizin Berlin, 10117 Berlin, Germany; frank.friedersdorff@charite.de

**Keywords:** CEUS, contrast-enhanced ultrasound, renal ultrasound, image quality, small renal mass (3–5)

## Abstract

Obesity and bowel gas are known to impair image quality in abdominal ultrasound (US). The present study aims at identifying individual factors in B-mode US that influence contrast-enhanced US (CEUS) image quality to optimize further imaging workup of incidentally detected focal renal masses. We retrospectively analyzed renal CEUS of focal renal masses ≤ 4 cm performed at our center in 143 patients between 2016 and 2020. Patient and lesion characteristics were tested for their influence on focal and overall image quality assessed by two experienced radiologists using Likert scales. Effects of significant variables were quantified by receiver operating characteristics (ROC) curve analysis with area under the curve (AUC), and combined effects were assessed by binary logistic regression. Shrunken kidney, kidney depth, lesion depth, lesion size, and exophytic lesion growth were found to influence focal renal lesion image quality, and all factors except lesion size also influenced overall image quality. Combination of all parameters except kidney depth best predicted good CEUS image quality showing an AUC of 0.91 (*p* < 0.001, 95%-CI 0.863–0.958). The B-mode US parameters investigated can identify patients expected to have good CEUS image quality and thus help select the most suitable contrast-enhanced imaging strategy for workup of renal lesions.

## 1. Introduction

Renal lesions are estimated to occur in 13% to 27% of the general population [1,2,3]. Small renal masses (SRMs) defined as lesions ≤ 4 cm, tend to be asymptomatic and are often detected incidentally on imaging [4,5]. It is generally known that the incidence of malignancy increases with the size of a SRM [6]. Therefore, early and accurate diagnosis of small renal lesions is very important to plan further patient management and ensure good patient outcome.

Since the risk of malignancy in solid renal tumors is high with incidences of 87.2% and 83.9% reported by Frank et al. and Kutikov et al., respectively [7,8], the choice of a suitable imaging method for reliable differentiation of malignant from benign lesions is essential for the diagnostic process. Often, a renal tumor is detected as an incidental finding in a routine ultrasound (US) examination, and the question as to the most appropriate further imaging strategy arises. Although US has many advantages including the absence of ionizing radiation as well as low costs and high availability, a systematic review by Vogel et al. identified poor diagnostic performance of conventional US in renal tumors [9], making contrast-enhanced imaging necessary for a reliable characterization. In this review, Vogel et al. showed comparable sensitivity for contrast-enhanced computed tomography (ceCT), contrast-enhanced magnetic resonance imaging (ceMRI) and contrast-enhanced ultrasound (CEUS) [9]. Furthermore, CEUS turned out to have higher diagnostic accuracy than ceCT in the evaluation of complex cystic renal masses [9].

Besides diagnostic accuracy, the setting of a contrast-enhanced examination plays a decisive role: while ceCT still remains the first-line imaging modality for SRMs, MRI has become more widely used over the last decade and also avoids radiation exposure, but its general use is limited by its availability and cost [10]. On the other hand, CEUS is superior regarding the evaluation of microcirculation as it uses a strictly intravascular contrast agent consisting of gas-filled microbubbles [10].

For CEUS of focal liver lesions, it has been shown that diagnostic confidence is improved by good examination conditions [11]. The authors of this study defined difficult ultrasound (US) conditions as the presence of meteorism, distinct steatosis, liver cirrhosis with inhomogeneous tissue, and obesity with a body mass index (BMI) > 30 kg/m^2^ [11].

In 2018, Sidhu et al. published the EFSUMB (European Federation of Societies for Ultrasound in Medicine and Biology) guidelines and recommendations for the use of CEUS in non-hepatic applications [12]. Next to renal ischemia, they identify focal renal lesions as the main indication for CEUS in the kidney. The focal renal lesions that can be diagnosed using CEUS are pseudotumors, cystic, indeterminate and solid masses as well as renal infections [12]. Thus, indications for CEUS include the whole range of SRMs investigated here, and the question arises of which patient-related imaging factors must be met to allow a CEUS examination likely to yield sufficient image quality for correct diagnostic characterization. This should help in deciding, in each case, whether a patient would benefit more from CEUS or cross-sectional imaging after initial sonographic detection of a SRM.

To our knowledge, this is the first study systematically analyzing essential patient and lesion characteristics and their influence on CEUS image quality in renal US.

## 2. Materials and Methods

This retrospective study was registered with our institution’s ethics committee (EA1/320/20). The oral and written informed consent of all patients was obtained before the examination. All study data were collected in compliance with the principles expressed in the 2002 Declaration of Helsinki.

### 2.1. Study Population

A database query for CEUS examinations of focal renal lesions performed in our hospital’s interdisciplinary ultrasound center between January 2016 and May 2020 was conducted. The cases retrieved by this search were screened regarding the following inclusion criteria: (I) age ≥ 18 years, (II) CEUS examination of a focal renal lesion ≤ 4 cm, and (III) sufficient image data for quality assessment (stored cine loops and multiple images). Exclusion criteria were (I) autosomal-dominant polycystic kidney disease and (II) no lesion or other indication for CEUS (assessment of renal perfusion).

### 2.2. CEUS Examination

Gray-scale B-mode US of the kidney was performed for lesion detection and for assessment of kidney size, echogenicity, and homogeneity using high-end ultrasound systems with a 1–6 MHz convex array transducer (Aplio i500/i900, Canon Medical Systems Corporation, Tochigi, Japan; Acuson Sequoia, Siemens Healthineers, Erlangen, Germany). The kidney was routinely examined in modified longitudinal and transverse planes and, if necessary, in deep inspiration and with optimized scanning positions.

CEUS examinations were performed during clinical routine using high-end ultrasound systems with up-to-date CEUS-specific protocols available at the time of the examination. The examinations were performed at 1–6 MHz with convex array transducers. A bolus of 1.6 mL of ultrasound contrast agent (SonoVue^®^, Bracco Imaging, Milan, Italy) was administered in all patients, and a very low mechanical index (MI < 0.1) was used to avoid early microbubble destruction. Penetration depth on CEUS was adapted by the investigator to clearly identify the target lesion and whole kidney. Baseline B-mode US and CEUS (for qualitative assessment of contrast enhancement pattern) were performed by a single highly experienced radiologist with more than ten years of experience in CEUS (EFSUMB level 3).

The associated data concerning the included patients were reviewed to collect individual information. The Radiology Information System (RIS) was used to cover age and gender.

### 2.3. Assessment of Image Quality

**Figure 1:**
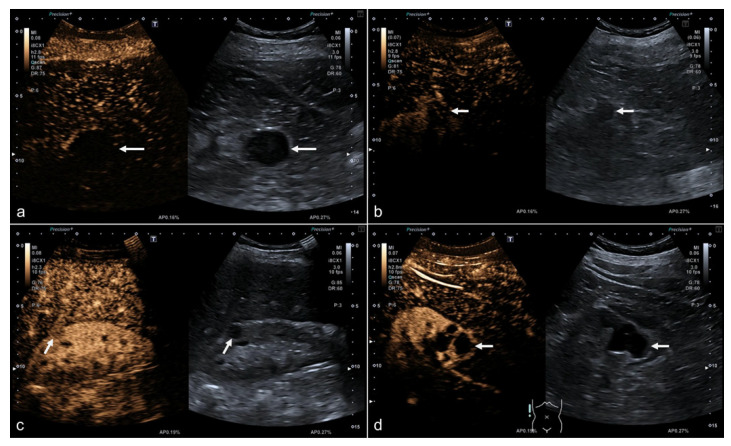
Examples of images illustrating different image quality scores. Images illustrating image quality of four different contrast-enhanced ultrasound (CEUS) examinations performed with the same US system, convex probe, and standardized imaging protocol (gain, dynamic range) with bolus injection of 1.6 mL SonoVue (Bracco Imaging): (**a**,**b**) Low image quality of a cystic and solid renal lesion, score of 1 for focal image quality in case of (**a**) and score of 2 for (**b**). (**c**,**d**) High image quality of a small solid lesion with a size of 12 mm (**c**) and an exophytic lesion at the lower pole of the kidney (**d**), both assigned scores of 5 for focal lesion.

Image quality was evaluated by two radiologists in consensus, one of them an EFSUMB level 3 examiner and both experienced in the field of renal CEUS. One factor assessed was presence of reduced parenchymal thickness or shrunken kidney (renal atrophy). Kidney depth and lesion depth were determined as the shortest distance of the renal capsule/superficial part of the lesion to the probe. Cases were stratified by lesion size and localization in the left versus right kidney and site within the kidney—upper third, middle or lower third—on representative CEUS loops, if not described in the diagnostic reports. Image quality at the target site (lesion) and overall image quality (kidney) were assessed in terms of diagnostic confidence by two experienced readers using an ordinal scoring system (Likert scale): 1—insufficient quality, 2—poor quality, 3—adequate quality, 4—good quality, 5—excellent quality. Representative examples of CEUS images illustrating different image qualities are shown in Figure 1.

### 2.4. Statistical Analysis

Continuous variables are reported as median and interquartile range (IQR) and categorical variables as absolute/total numbers (n/N) and percentages in brackets. The aim of our analysis is to identify patient and lesion factors that affect CEUS image quality. Therefore, image quality scores—ordinally scaled—were correlated with the presence of various patient- and lesion-related variables using the Chi^2^ test for variables measured in ratio scale and the Kruskal-Wallis test for ordinally scaled variables. Effects were analyzed for both focal (site of renal lesion) and overall image quality. In addition, a univariate ANOVA was performed to detect possible uncertainties. Moreover, post hoc testing with Bonferroni correction was done to ensure that at least two image quality groups differed statistically significantly from each other. To investigate the interdependence of impact of lesion location (right vs. left kidney and kidney third) on image quality, a two-factorial ANOVA of these two factors was performed.

For the parameters identified to have a statistically significant influence on image quality, the effect was quantified by receiver operating characteristics (ROC) curve analysis with quantification of the area under the curve (AUC). Therefore, good image quality was defined as a score of 4 or 5 on the Likert scale as described above. Furthermore, different combinations of individual parameters with a statistically significant influence on image quality were tested by binary logistic regression to determine the AUCs quantifying the influence of the combined parameters. The best combination of individual parameters was identified as the combination with the largest AUC and the smallest number of included parameters compared to other combinations with the same AUC.

A two-sided significance level of α = 0.05 was considered appropriate to indicate statistical significance. All statistical analyses were performed using the SPSS software (IBM Corp. Released 2019. IBM SPSS Statistics for Windows, Version 26.0. IBM Corp: Armonk, NY, USA.).

## 3. Results

### 3.1. Study Population

The final study population included 143 patients with at least one renal target lesion ≤ 4 cm and sufficient stored image data for quality assessment. The patients’ baseline characteristics are presented in Table 1. Ninety-four of the initially identified CEUS examinations were excluded since they were repeat follow-up examinations of already included patients. The study patients had a median age of 62 years (IQR, 52–75 years). Cystic renal lesions were found in 78.3% of the cases and 21.7% as solid renal lesions. Overall mean lesion depth was 61 mm (IQR, 46–74 mm).

Presented are the baseline characteristics of the study population subdivided into patient- and lesion-related features. Continuous variables are given as median (IQR), categorical variables as absolute/total numbers (n/N) and percentages in brackets.

### 3.2. Assessment of Image Quality

**Figure 2:**
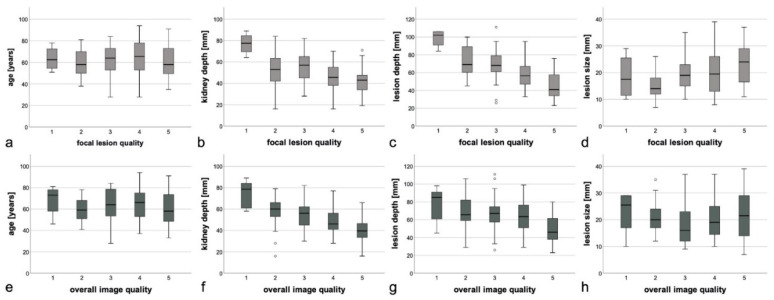
Distribution of continuous variables age, kidney depth, lesion depth and lesion size in focal and overall image quality classes. Boxplots of the distributions of the continuous variables across the five image quality classes (Likert scores) for focal image quality (**a**–**d**) and overall image quality (**e**–**h**). The results of the statistical tests are outlined in Table 2. (**a**,**e**) The age of the patient showed, neither for focal nor for overall image quality, a statistically significant relationship which could be visualized using boxplots.(**b**,**f**) The kidney depth showed for focal image quality, as well as overall image quality lower mean kidney depth for higher image quality.(**c**,**g**) The same relationship as described for kidney depth (**b**,**f**) applies for lesion depth and image quality. (**d**,**h**) The lesion size shows higher mean lesion size for higher focal image quality. For overall image quality, no tendency is visible.

Arithmetic means of image quality scores were 3.7 and 3.6 for focal and overall image quality, respectively. Focal image quality scores 1–5 were distributed as follows: 4 (2.8%), 15 (10.5%), 43 (30.1%), 42 (29.4%), and 39 (27.3%). For overall image quality, score distribution was: 6 (4.2%), 22 (15.4%), 39 (27.3%), 28 (19.6%), and 48 (33.6%). Correlation with individual patient and lesion characteristics yielded the following results: there were no strong correlations between imaging quality and age, sex, reduced cortical thickness or entity, localization, and size of lesion (Table 2). A statistically significant increase in image quality was found for (I) exophytic growth of focal renal lesion, (II) absence of shrunken kidneys, (III) lower lesion depth, and (IV) lower depth of lesion-bearing kidney (Table 2, Figure 2). For intrarenal lesion site (upper, middle, lower third), the Chi^2^ test yielded no correlation with image quality (*p* = 0.064), whereas ANOVA reached significance (*p* = 0.040). With the restrictive approach used here, we do not interpret the results as showing a strong correlation in order to satisfy the discrepancy between the two applied statistical tests.

Table 2 presents the results of the statistical tests investigating effects on focal and overall image quality. For each variable and both focal and overall image quality, a nonparametric test and an ANOVA were performed to account for possible uncertainties.

The two-sided ANOVA confirmed the results given in Table 2, showing both focal lesion quality (*p* = 0.155) and overall quality (*p* = 0.127) not to be impacted by the combined lesion location parameters (right/left kidney and intrarenal lesion site).

### 3.3. Post Hoc Tests

The results of post hoc ANOVA confirmed that sex, age, lesion type, shrunken kidney, and side of involved kidney had no statistically significant impact on focal or overall image quality. Additionally, intrarenal lesion localization (third) was shown to have no statistically significant effect on focal or overall image quality, whereas univariate ANOVA of focal lesion quality identified an effect of intrarenal lesion localization (*p* = 0.046), which was confirmed by the Chi^2^ test (*p* = 0.064). As expected from univariate ANOVA, testing with Bonferroni correction also identified no statistically significant effect of lesion size on overall image quality.

For both focal and overall image quality, statistically significant (*p* ≤ 0.05) differences between at least two groups were found in groupwise comparisons of image quality performed with Bonferroni correction for the following parameters: shrunken kidney, kidney depth, lesion depth, and exophytic lesion growth. For lesion size, a statistically significant difference between at least two groups was found only for the effect on focal image quality.

### 3.4. ROC Analysis

**Figure 3:**
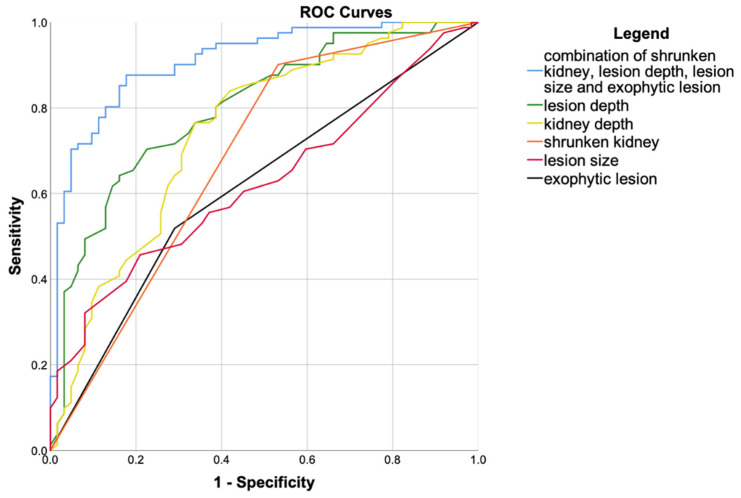
ROC curves of the single parameters and the best combination.

ROC analysis was performed to quantify the characteristic’s influence on reaching high image quality (≥4 Likert scores). The results of ROC curve analysis with the area under the curve (AUC) for continuous and categorial variables are presented in Table 3 and in Figure 3.

ROC analysis was performed to quantify effects of statistically significant variables influencing image quality (Table 2). The ROC curves of all variables showed statistical significance. Nevertheless, the asymptotic 95%-CI of exophytic lesion growth strikes 0.5 in overall image quality and was therefore not considered in further evaluation.

Presented are receiver operating characteristics (ROC) curves of individual parameters and the best combination, as presented in Table 4, influencing focal image quality (Likert score of 4 as cut-off). Diagonal segments were produced by ties.

AUC revealed lesion depth to be associated with focal image quality and kidney depth to be the strongest predictor of overall image quality, confirming the theoretical expectation regarding image quality assessment.

### 3.5. Combined ROC Analysis

As described in the Methods section, the combination of shrunken kidney, lesion depth, lesion size, and exophytic lesion growth were identified to be the most suitable combination of parameters (Table 4) showing strong correlation with good focal image quality (score of 4 or 5). with an effect size of an AUC of 0.91 (asymptotic 95%-CI: 0.863–0.958) and asymptotic statistical significance of *p* < 0.001.

## 4. Discussion

The major results of the present study can be summarized as follows: (I) CEUS image quality is reduced in shrunken kidneys and improved when examining exophytically growing lesions, and with shorter distance of the kidney and the lesion from the transducer; this applies to both focal and overall image quality; (II) focal, but not overall, image quality increases with lesion size, while patient age and sex, lesion entity, reduced parenchymal thickness and lesion localization do not impact CEUS image quality; and (III) the significant parameters just mentioned above improve focal image quality more markedly than the individual parameters alone, with the combination of shrunken kidney, lesion depth, lesion size, and exophytic lesion growth proving to be the most suitable combination.

Putz et al. reported meteorism and obesity as the main patient-related factors with a negative effect on CEUS image quality [11]. While CEUS is predominantly used for liver imaging, renal applications of CEUS have attracted growing interest. Therefore, an interest exists in knowing which patient factors might reliably predict a sufficient CEUS image quality. This is the rationale for our study, which—to our knowledge—is the first systematic analysis of individual patient- and lesion-related factors that have an effect on the image quality of renal CEUS.

Nevertheless, it must be mentioned that not only patient and lesion characteristics influence image quality, and therefore diagnostic accuracy, but also artifacts which are partially CEUS-specific, such as near-field signal loss due to microbubble destruction—which can be influenced by using a specific configuration of the US machine [13,14].

Our results have important implications for the diagnostic workup of SRMs detected on nonenhanced imaging: CEUS shows high diagnostic performance [9] and other advantages including a low rate of side effects [15]. Therefore, CEUS is generally preferred for the characterization of focal renal lesion. A recently published study showed CEUS, even in a small cohort of six pregnant women, to be a safe imaging tool [16]. Knowing beforehand whether a chosen imaging modality is likely to yield a diagnosis can shorten the diagnostic process, improving patient comfort and outcome. Using CEUS only where it is expected to achieve diagnostic quality, its instantaneous diagnosis determines directly if cross-sectional imaging is necessary for cancer staging if a malignant lesion is diagnosed, thus preventing unnecessary imaging in patients with benign lesions.

The prediction as to whether CEUS or MRI might be the better imaging method for further characterization of an SRM incidentally detected by plain B-mode ultrasound also has important economic implications, identifying patients not in need of undergoing MRI.

CEUS benefits—especially shown for renal cysts—from a higher temporal resolution than CT and MRI, allowing real-time evaluation of the enhancement pattern [17,18,19].

Therefore, immediate workup of an incidental SRM by CEUS in suitable patients can save costs by replacing cross-sectional MRI. Besides, the MRI and CT slots not needed for patients worked up by CEUS can help other patients to obtain their MRI or CT examination more quickly.

Apart from what has been discussed so far, patient preferences should also play a role in selecting an imaging modality. For example, Thorpe et al. found that more than 50% of individuals have a high grade of anxiety during an MRI examination, which could, for instance, promote the occurrence of motion artifacts [20]. Another concern with ceMRI is that gadolinium deposition in the brain has been observed in patients undergoing repeated MRI with administration of a gadolinium-based contrast agent—although its pathologic value is unclear [21,22]. Nevertheless, clinically relevant side effects of iodinated contrast agents used in ceCT are more common: “contrast-induced nephropathy” or “postcontrast acute kidney injury” has an incidence between 5.0% and 6.4% based on meta-analysis data [23,24,25]. Not being limited by adverse effects such as nephrotoxicity, cumulative radiation exposure or gadolinium deposition, CEUS is well suited for long-term surveillance, for instance, in patients with Bosniak IIF cysts [26].

Besides the image quality expectable in CEUS examination, it must be mentioned that patients with shrunken kidneys might not provide a high image quality, but would suffer from iodinated contrast agents in ceCT, since impaired renal function was found to be associated with contrast-induced nephropathy [24]. Although, as mentioned above, its clinical relevance is subject to controversial discussions, impaired renal function also leads to a reduced elimination of Gadolinium-containing contrast agents used in ceMRI [27]. So an expected low image quality could be relativized by potential harm using the alternative of contrast-enhanced imaging.

Experienced examiners are able to estimate CEUS image quality from B-mode image quality. Nevertheless, our results can help less experienced examiners and allow an objective assessment of expected CEUS image quality in inconclusive cases. Moreover, our approach is straightforward, using criteria that are rapidly assessed such as lesion depth or exophytic lesion growth. Although the two variables show comparable results in our study, lesion depth should be preferred to kidney depth, since this information is also important for lesion characterization rather than for assessing obesity only. Finally, the parameters presented here should be considered together, since the AUC for individual parameters alone are not larger than 0.8 (Table 3). Obviously, an experienced examiner can also characterize SRMs with a lower image quality, but we used high-end US-devices in our study and acquisition of CEUS-loops by an experienced radiologist and vindicate, therefore, our ROC analyses (Table 3 and Table 4, Figure 3) with an image quality score of four as cut-off.

### 4.1. Limitations

Our study is limited by its retrospective and single-center design. Nevertheless, all patients were examined with an identical CEUS protocol. All ultrasound examinations were performed using high-end systems with state-of-the-art CEUS-specific protocols, resulting in generally high image quality. Nevertheless, we compared CEUS loops obtained with a standardized protocol to assess image quality and imaging parameters, and not the image quality of the system as such.

### 4.2. Conclusions

Focal image quality of CEUS examinations is impaired by shrunken kidney, a large distance of the kidney and lesion from the body surface, and smaller lesion size, while exophytic growth of a focal renal lesion results in better image quality. Awareness of patient and lesion factors that degrade image quality can be used for better patient selection and can thus improve diagnostic confidence of examiners performing CEUS.

## Figures and Tables

**Table 1:** Baseline characteristics of included patients.

Variable	Value
Characteristics of the patients	
Age [years]	62 (52–75)
Female sex	43/143 (30.1%)
Characteristics of the kidney	
Kidney depth [mm]	48 (39–62)
Shrunken kidney	37/143 (25.9%)
Reduced cortical thickness	40/143 (28.0%)
Characteristics of the lesion	
Cystic	112/143 (78.3%)
Solid	31/143 (21.7%)
Depth of renal lesion [mm]	61 (46–74)
Largest lesion diameter [mm]	20 (14–26)
Left side	72/143 (50.3%)
Right side	71/143 (49.7%)
Upper third	45/143 (31.5%)
Middle third	65/143 (45.5%)
Lower third	33/143 (23.1%)
Exophytic lesion growth	60/143 (42.0%)

Abbreviations: IQR denotes interquartile range.

**Table 2:** Influence of patient and lesion characteristics on focal and overall image quality.

Variable	Focal Quality		Overall Quality	
	Nonparametric test	ANOVA	Nonparametric test	ANOVA
Age	0.750 ^2^	0.809	0.387 ^2^	0.460
Sex	0.290 ^1^	0.296	0.426 ^1^	0.434
Entity ^3^	0.433 ^1^	0.441	0.134 ^1^	0.135
Reduced parenchymal thickness	0.807 ^1^	0.814	0.275 ^1^	0.280
Shrunken kidney	<0.001 ^1^	<0.001	<0.001 ^1^	<0.001
Kidney depth	<0.001 ^2^	<0.001	<0.001 ^2^	<0.001
Lesion depth	<0.001 ^2^	<0.001	<0.001 ^2^	<0.001
Lesion size	0.006 ^2^	0.004	0.385 ^2^	0.494
Exophytic lesion growth	0.043 ^1^	0.042	0.021 ^1^	0.020
Side	0.321 ^1^	0.328	0.923 ^1^	0.926
Intrarenal third	0.064 ^1^	0.040	0.156 ^1^	0.211

^1^ tested with the Chi^2^ test, ^2^ tested with Kruskal-Wallis test; ^3^ entity was stratified as cystic versus solid lesion; ANOVA denotes analysis of variance.

**Table 3:** ROC analysis to quantify effects of variables predicting high image quality.

Variable	Focal Quality			Overall Quality		
	AUC	Asymptotic significance	Asymptotic 95%-CI	AUC	Asymptotic significance	Asymptotic 95%-CI
Shrunken kidney	0.684	<0.001	0.593–0.776	0.748	<0.001	0.664–0.832
Kidney depth	0.744	<0.001	0.661–0.827	0.776	<0.001	0.696–0.856
Lesion depth	0.800	<0.001	0.727–0.873	0.695	<0.001	0.609–0.781
Lesion size	0.625	0.011	0.534–0.715	–	–	–
Exophytic lesion growth	0.614	0.020	0.521–0.707	0.502	0.049	0.406–0.597

ROC denotes receiver operating characteristics, AUC denotes area under the curve, CI denotes confidence interval.

**Table 4:** ROC analysis of combined variables and their effect in predicting high (score of 4 or 5) focal image quality.

No.	Combined Variables					ROC Analysis		
	Shrunken kidney	Kidney depth	Lesion depth	Lesion size	Exophytic lesion growth	AUC	Asymptotic significance	Asymptotic 95%-CI
1		X	X			0.812	<0.001	0.741–0.883
2	X	X	X			0.863	<0.001	0.805–0.921
3	X				X	0.773	<0.001	0.697–0.850
4			X	X	X	0.843	<0.001	0.777–0.909
5			X	X		0.834	<0.001	0.767–0.902
6	X		X	X		0.893	<0.001	0.841–0.945
7		X	X	X	X	0.851	<0.001	0.787–0.915
8	X	X		X	X	0.870	<0.001	0.812–0.928
9	X		X	X	X	0.910	<0.001	0.863–0.958
10	X	X	X	X	X	0.910	<0.001	0.863–0.957
11		X	X	X		0.842	<0.001	0.777–0.907

Since all variables were quantified regarding effect on high focal image quality (score of 4 or 5), eleven different combinations were investigated. With each of them, a bivariate logistic regression and ROC analysis were performed. Presented are the combinations, with ‘X’ indicating the single parameters to participate in the bivariate logistic regression and their AUC with asymptotic significance and 95%-CI. Combination No. 9 generates the largest AUC, while including one variable less than combination No. 10. ROC denotes receiver operating characteristics, AUC denotes area under the curve, CI denotes confidence interval.

## Abbreviations

ANOVA	analysis of variance
AUC	area under the curve
BMI	body mass index
CEUS	contrast-enhanced ultrasound
ceCT	contrast-enhanced computed tomography
ceMRI	contrast-enhanced magnetic resonance imaging
CI	confidence interval
IQR	interquartile range
ROC	receiver operating characteristics
SRM	small renal mass
US	ultrasound
